# Preliminary Study on Synergistic Effects of Humic Acid and Seaweed Extract on Cereal Crop Yield and Competitiveness with Wild Weed Beets (*Beta vulgaris* L.)

**DOI:** 10.3390/plants14243770

**Published:** 2025-12-11

**Authors:** Zainulabdeen Kh. Al-Musawi, Husam S. M. Khalaf, Ali A. Hassouni, Rusul R. Shakir, Viktória Vona, István Mihály Kulmány

**Affiliations:** 1 Agricultural and Food Research Centre, Széchenyi István University, 9026 Győr, Hungary; kulmany.istvan@sze.hu; 2 Department of Plant Sciences, Albert Kázmér Faculty of Agricultural and Food Sciences in Mosonmagyaróvár, Széchenyi István University, 9200 Mosonmagyaróvár, Hungary; 3 Department of Field Crops, College of Agriculture, Al-Muthanna University, Samawah 6601, Iraq; husam.saadi84@gmail.com; 4 College of Agriculture, Misan University, Misan 62001, Iraq; 5 Department of Agronomy and Soil Science, School of Environmental and Rural Sciences, University of New England, Armidale, NSW 2350, Australia; rusul.shakir91@gmail.com; 6 Department of Water Management and Natural Ecosystems, Albert Kázmér Faculty of Agricultural and Food Sciences in Mosonmagyaróvár, Széchenyi István University, 9200 Mosonmagyaróvár, Hungary; 7 HUN-REN-SZE PhatoPlant-Lab, Albert Kázmér Faculty of Agricultural and Food Sciences, Széchenyi István University, 9200 Mosonmagyaróvár, Hungary

**Keywords:** humic acid, seaweed extract, wheat (*Triticum aestivum*), barley (*Hordeum vulgare*), oat (*Avena sativa*), crop–weed competition, sustainable agriculture

## Abstract

Crop–weed competition markedly reduces cereal yield. Integrative weed management approaches, involving the use of humic acid (HA) and seaweed extract (SWE), have gained attention as herbicide efficacy declines and environmental concerns grow. However, potential synergistic effects between HA and SWE have not yet been investigated. We evaluated the effects of HA, SWE, and their combination (HA+SWE) on the growth, yield, and competitive ability of cereals against wild weed beets (*Beta vulgaris* L.). A single-season field experiment was conducted using a split-plot design within a randomised complete block to assess the effects of treatment amendments on wheat, barley, and oats. The results showed that HA and HA+SWE organic amendments consistently improved grain yield and biomass across crop species. SWE responses varied across species, indicating species-dependent sensitivity. In addition, HA enhanced barley weed suppression, highlighting its dual roles in improving crop vigour and reducing weed proliferation. In contrast, SWE modestly increased spike length in oats, emphasising its effect on crop growth characteristics. Overall, these preliminary findings support targeted biostimulant use to enhance cereal yield and integrate weed management into sustainable cropping systems.

## 1. Introduction

Cereals such as wheat (*Triticum aestivum* L.), barley (*Hordeum vulgare* L.) and oat (*Avena sativa* L.) are of major economic and nutritional value [1,2]. As staple food crops, they provide > 50% of human caloric intake and serve as animal feed and industrial raw materials. However, their productivity is strongly limited by crop–weed competition [3,4].

Wild weed beets (*Beta* sp.) are derived from self-domestication, hybridisation with sea beets [*Beta vulgaris* subsp. *maritima* (L.) Arcang.], or the cultivation of sugar beets (*Beta vulgaris* L.) [5,6]. Recently, they have been extensively distributed across European sugar beet fields due to hybridisation between cultivated sugar beets and their wild relatives. Gene flow has resulted in feral hybrids with enhanced competitiveness, making wild weed beets more problematic and difficult to control in cereal–beet rotations [7,8,9].

Weed infestation remains a major challenge in cropping systems, especially cereals, where weeds compete for light, nutrients, water, and space, often causing substantial yield losses. Estimated cereal yield reductions due to weed interference range from approximately 26% to 40%, and may be higher under severe pressure [10,11,12]. Moreover, several dominant weed species associated with sugar beet production have evolved resistance to commonly used herbicides, thereby reducing the effectiveness of chemical control [13,14]. Concerns also persist regarding the long-term impacts of herbicide application on sustainability and food safety, as the overuse of herbicides contributes to herbicide resistance [15], soil biodiversity loss, water contamination, and increased production costs when the environmental externalities are considered [16,17]. Similar trends have been recorded in other cropping systems, where herbicides exert only a minor impact on weed community structure compared with environmental and cultural factors, highlighting the limitations of management strategies based on herbicides [18]. Therefore, there is increasing interest in non-chemical and eco-friendly weed management strategies [19,20], such as using organic amendments [e.g., humic acid (HA) and seaweed extract (SWE)] to improve crop competitiveness and, thus, address the environmental and economic problems caused by weed infestation [19,20].

HA is a naturally occurring polymer and heterocyclic compound with biostimulant and soil-conditioning properties [21,22]. It plays an essential role in enhancing plant growth and stress tolerance, as evidenced by its ability to increase plant dry biomass and improve crop resistance to environmental stressors [23,24,25]. Moreover, HA application in cereals such as maize has been shown to increase grain yield by enhancing the uptake of essential nutrients, including nitrogen, phosphorus and potassium [26,27,28,29], thereby improving crop growth [30,31]. Furthermore, a recent meta-analysis reported that HA application can increase crop production by approximately 12% and nitrogen use efficiency by 27% across various agroecological zones, highlighting its important role in sustainable agriculture [32]. Although HA is widely used in intensive technologies to enhance the physiological potential of crops [33,34,35,36], it elicits a restrictive effect on crop genotypes [36,37] because it is not a universal agent that promotes the occurrence of novel, non-inherent plant growth characteristics [36]. By modulating hormone-like activity, photosynthesis and respiration, the carboxyl and phenolic groups in HA enhance nutrient availability, water retention, microbial activity and stress tolerance at the molecular and edaphic levels [34,35,38].

Aside from promoting crop growth, previous studies have reported that HA is instrumental in enhancing soil physical and biochemical properties. It improves soil structure, texture, water-holding capacity and microbial activity, increases macro- and micronutrient availability [34,38,39,40,41], and reduces the mobility of toxic heavy metals in the soil [38,42]. However, the effects of HA stimulants on crop performance and soil conditions remain inconsistent [38,43,44,45,46] largely due to conflicting research findings across varying HA doses and soil characteristics. For instance, Rose et al. [24] reported that HA application influenced soil chemical characteristics; however, its effects on crop growth traits remain uncertain. Meanwhile, Gollenbeek and Van Der Weide [47] found that HA application improved soil physical properties.

Furthermore, HA may indirectly suppress weed proliferation by increasing crop vigour and canopy closure, reducing competitive ecological niches and inducing allopathic shifts in soil microbial communities in favour of crops [31,36]. Canopy formation and other competitive characteristics of crops play an important role in reducing weed biomass by quickly closing the canopy, thereby restricting the access of weeds to light [19]. Moreover, field experiments revealed that humic preparations, especially when combined with herbicides or nitrogen-based fertilisers, may improve crop yield and enhance weed control efficacy, as in the case of wheat [36].

As with HA, previous studies have shown that the method and frequency of SWE application can significantly influence crop growth responses [48,49,50]. Seaweeds are macroalgae that are integral components of marine and coastal ecosystems; they contribute to marine biodiversity and the biosphere [51]. Seaweeds are rich in phytohormones (e.g., auxins, cytokinins and gibberellins), polysaccharides, amino acids, and minerals that regulate germination, root growth, chlorophyll biosynthesis and photosynthesis. Consequently, SWE enhances plant tolerance to both abiotic (e.g., drought, salinity and temperature) and biotic (e.g., fungal, bacterial and viral pathogens) stressors [48,51,52]. SWE has been documented to improve crop performance in terms of germination, seed vigour, biomass, and yield quality in several crop species, including tomato (*Solanum lycopersicum* L.), sweet pepper (*Capsicum annuum* L.), lettuce (*Lactuca sativa* L.), cucumber (*Cucumis sativus* L.), strawberry (*Fragaria × ananassa* (Duchesne ex Weston) Duchesne ex Rozier), grapevine (*Vitis vinifera* L.) and kiwifruit (*Actinidia deliciosa* L.) [51,52,53,54]. However, SWE responses are species- and context-specific, and benefits are often more pronounced when combined with nitrogen fertilisers [55,56,57].

Although HA and SWE are being considered in sustainable farming, their combined effects on cereal competitiveness against wild weed beets, especially under the semi-arid conditions of southern Iraq, have not yet been investigated. Integrating HA and SWE could offer complementary advantages as HA mainly improves soil productivity and root growth, while SWE accelerates shoot enlargement and accentuates reproductive characteristics. For instance, it has been recently reported that the foliar application of a combination of HA and SWE on fruit crops grown in arid environments can greatly improve their nutrient uptake, yield and quality [58,59]. Meanwhile, the preliminary results of the combined application of HA and SWE to barley suggest synergistic effects; however, comprehensive multi-environment trials validating these findings are still lacking [60].

Hence, this study was conducted to evaluate the synergistic effects of HA and SWE on the growth, yield, and weed-suppression ability of three cereal crops grown in competition with wild weed beets. Specifically, the objectives were to determine (i) species-specific reactions of wheat, barley and oats to HA, SWE and HA+SWE organic amendment; (ii) weed suppression dynamics based on the responses of aggressive wild weed beets to biostimulants; (iii) synergistic interactions between HA and SWE; and (iv) biostimulant effects under semi-arid conditions. The overall aim was to evaluate the combined effects of humic acid and seaweed extract on cereal crop performance and weed suppression to support the development of species-specific amendment strategies to enhance crop competitiveness, yield, and quality.

## 2. Results

### 2.1. Effects of Organic Amendments on Spike Length

**Figure 1:**
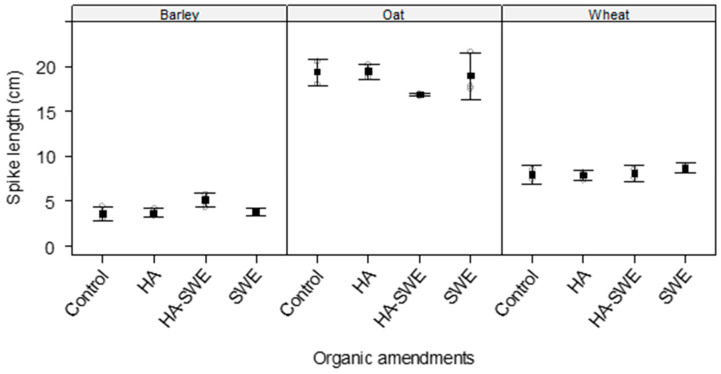
Effect of humic acid (HA), seaweed extract (SWE), and a mixture of HA and SWE (HA+SWE) on the spike length of cereal crops. The black squares represent the mean spike length (cm) of the crops under each organic amendment. The vertical bars represent a 95% confidence interval. The hollow circles represent the replicates. Data were collected at the conclusion of the 2021–2022 winter growing season experiment. Each mean value represents *n* = 3 replicates per treatment × crop species.

The two-way analysis of variance (ANOVA) revealed that the effects of organic amendments on the spike length of the cereal crops were not significant (F_3,18_ = 1.78, *p* = 0.197; Table 1), indicating consistent results across replicates (Figure 1).

In contrast, highly significant differences were observed among the crop species (F_2,18_ = 615.70, *p* < 0.000001; Table 1). Across all treatments, oats had the longest spikes (18.65 cm), whereas barley had the shortest (4.11 cm; Figure 1).

The results also showed a significant interaction between crop species and treatments (F_6,18_ = 2.76, *p* = 0.0443; Table 1), suggesting that the response of spike length to organic amendments varied by cereal species. For instance, the spikes of oats were longest under the HA organic amendment (19.47 cm), followed by the SWE (19.0 cm) and HA+SWE organic amendment (16.85 cm). However, barley had the tallest spikes under the combined amendment (5.12 cm) compared with that of the others organic amendments, but it had overall the shortest spikes across organic amendments in comparison with the other crop species (Figure 1).

### 2.2. Effects of Organic Amendments on Spike Number

The results of ANOVA revealed that the organic amendments had highly significant effects on the spike number of the cereal crops (F_3,18_ = 5.32, *p* = 0.00838; Table 1). The HA+SWE organic amendment produced the highest spike number (1109 spikes m^−2^), followed by the SWE organic amendment (914 spikes m^−2^). In contrast, spike number did not significantly differ among the crop species (F_2,18_ = 0.85, *p* = 0.44; Table 1).

**Figure 2:**
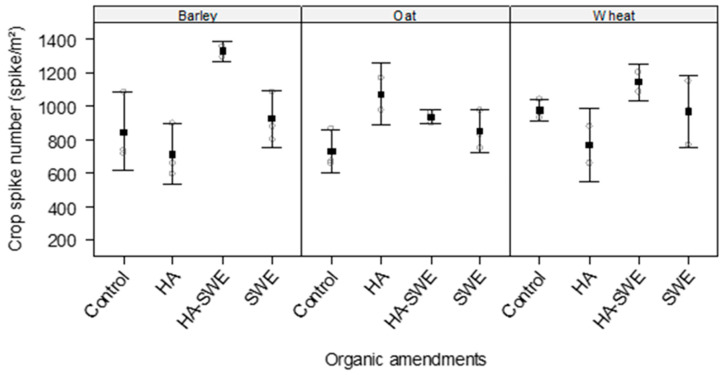
Effect of humic acid (HA), seaweed extract (SWE), and a mixture of HA and SWE (HA+SWE) on the spike number of cereal crops. The black squares represent the mean spike number (spikes m^−2^) of cereal crops under each treatment. The vertical bars represent a 95% confidence interval. The hollow circles represent the replicates. Data were collected at the conclusion of the 2021–2022 winter growing season experiment. Each mean value represents *n* = 3 replicates per treatment × crop species.

Nonetheless, a significant interaction between crop species and treatments (F_6,18_ = 3.41, *p* = 0.020) was observed (Table 1), indicating that the response of spike number to organic amendments varied by cereal species (Figure 2). The spike number of barley (1328 spike m^−2^) and wheat (1144 spikes m^−2^) was highest under the HA+SWE organic amendment, whereas that of oats was highest under the HA organic amendment (1072 spikes m^−2^).

### 2.3. Effects of Organic Amendments on Kernel Weight

**Figure 3:**
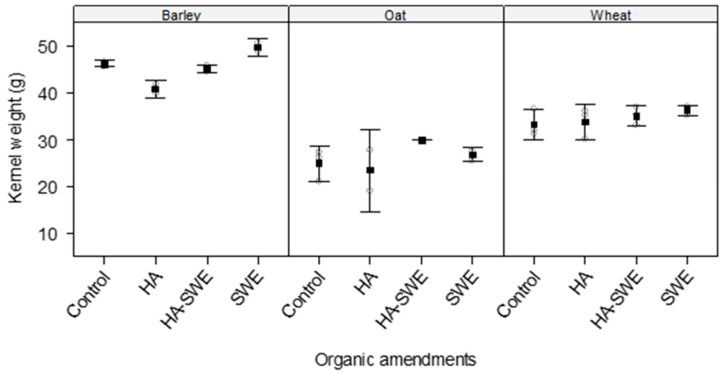
Effect of humic acid (HA), seaweed extract (SWE), and a mixture of HA and SWE (HA+SWE) on the kernel weight of cereal crops. The black squares represent the mean kernel weight (g) of cereal crops under each treatment. The vertical bars represent a 95% confidence interval. The hollow circles represent the replicates. Data were collected at the conclusion of the 2021–2022 winter growing season experiment. Each mean value represents *n* = 3 replicates per treatment × crop species.

The results of ANOVA revealed highly significant differences (F_3,18_ = 6.09, *p* = 0.00479) among the organic amendments in terms of kernel weight (Table 1). Kernel weight was highest under the HA+SWE organic amendment (37.5 g), whereas the lowest occurred under the HA organic amendment (32.8 g; Figure 3).

Similarly, crop species had a strong influence on kernel weight (F_2,18_ = 187.98, *p* < 0.000001; Table 1), with barley producing the greatest kernel weight across the treatments (Figure 3).

Additionally, the interaction between crop species and the organic amendment treatments was significant (F_6,18_ = 2.67, *p* = 0.04945), demonstrating that the response of kernel weight to the biostimulants varied by crop species (Table 1). Under the SWE and HA+SWE organic amendment, barley displayed the greatest kernel weight (49.4 and 45.0 g, respectively), followed by wheat (36.2 and 35.0 g, respectively; Figure 3). Although the SWE organic amendment increased the kernel mass of barley by 7%, it had a limited effect on wheat.

### 2.4. Effects of Organic Amendments on Grain Yield

**Figure 4:**
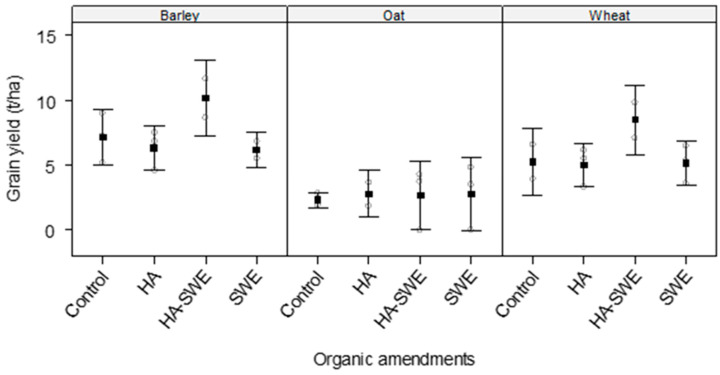
Effect of humic acid (HA), seaweed extract (SWE), and a mixture of HA and SWE (HA+SWE) on the grain yield of cereal crops. The black squares represent the mean grain yield (t ha^−1^) of cereal crops under each treatment. The vertical bars represent a 95% confidence interval. The hollow circles represent the replicates. Data were collected at the conclusion of the 2021–2022 winter growing season experiment. Each mean value represents *n* = 3 replicates per treatment × crop species.

The results of ANOVA revealed highly significant differences (F_3,18_ = 6.88, *p* = 0.00389) among the organic amendments in terms of grain yield (Table 1). Grain yield was highest under the HA+SWE organic amendment (6.49 t ha^−1^), showing an approximately 32.7% increase compared with that of the control; in contrast, it was lowest under the SWE organic amendment, regardless of crop species (Figure 4).

The results of ANOVA also revealed that the crop species elicited an extremely strong effect on grain yield (F_2,18_ = 24.67, *p* < 0.0001). Among the crop species, barley produced the greatest grain yield (7.3 t·ha^−1^), whereas oats produced the lowest grain yield (2.65 t ha^−1^; Figure 4).

However, the interaction between crop species and organic amendments was not significant (F_6,18_ = 0.57, *p* = 0.74784), suggesting that yield improvements conferred by the amendments were broadly similar across the cereal crops (Figure 4). Compared with the control, the HA and SWE organic amendments increased the grain yield of oats by approximately 14–19%, whereas the HA+SWE organic amendment increased the grain yield of barley and wheat by 42% and 60%, respectively.

### 2.5. Effects of Organic Amendments on Aboveground Crop Biomass

**Figure 5:**
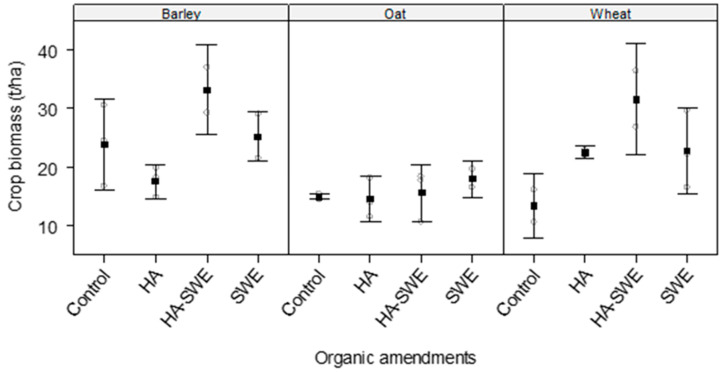
Effect of humic acid (HA), seaweed extract (SWE), and a mixture of HA and SWE (HA+SWE) on the aboveground biomass of cereal crops. The black squares represent the mean biomass (t ha^−1^) of cereal crops under each treatment. The vertical bars represent a 95% confidence interval. The hollow circles represent the replicates. Data were collected at the conclusion of the 2021–2022 winter growing season experiment. Each mean value represents *n* = 3 replicates per treatment × crop species.

The results showed that the organic amendments significantly influenced aboveground crop biomass (F_3,18_ = 4.56, *p* = 0.016) compared with that of the control (Table 1). Biomass was greatest under the HA+SWE organic amendment (25.20 t ha^−1^; Figure 5).

Similarly, crop species exerted a highly significant effect on aboveground crop biomass (F_2,18_ = 10.02, *p* = 0.00133), reflecting substantial differences in biomass accumulation among the three cereal crops (Table 1). Barley (24.25 t ha^−1^) produced the greatest biomass, followed by wheat (22.61 t ha^−1^) and oats (15.57 t ha^−1^; Figure 5).

The interaction between crop species and treatment was not significant (F_6,18_ = 2.38, *p* = 0.07512), suggesting species-specific responses to amendment type (Figure 5). Under the HA organic amendment, wheat produced the greatest biomass, followed by barley and oats. Meanwhile, under the SWE and HA+SWE organic amendment, barley produced the greatest biomass, whereas oats produced the lowest biomass (Figure 5).

### 2.6. Effects of Organic Amendments on the Biomass of Wild Weed Beets

**Figure 6:**
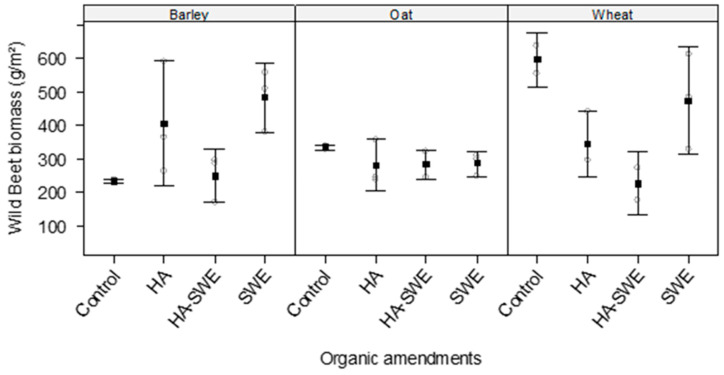
Effect of humic acid (HA), seaweed extract (SWE), and a mixture of HA and SWE (HA+SWE) on the biomass of wild weed beets (g m^−2^). The black squares represent the mean dry weight of wild weed beets in competition with cereal crops under each treatment. The vertical bars represent a 95% confidence interval. The hollow circles represent the replicates. Data were collected at the conclusion of the 2021–2022 winter growing season experiment. Each mean value represents *n* = 3 replicates per treatment × crop species.

The results of ANOVA revealed that the organic amendments (F_3,18_ = 5.63, *p* = 0.0067) significantly affected the dry weight of wild weed beets (Table 1). Specifically, it decreased to 256 g m^−2^ under the HA+SWE organic amendment compared with that of the other organic amendment treatments (Figure 6).

The results also showed that the crop species significantly differed in their effects on the dry weight of wild weed beets (F_2,18_ = 5.50, *p* = 0.01365). The highest dry weight of wild weed beets was recorded in the wheat plots (410.42 g m^−2^), whereas the lowest was recorded in the oat plots (291.84 g m^−2^).

Similarly, the interaction between cereal crop species and organic amendments was highly significant (F_6,18_ = 5.19, *p* = 0.00294; Table 1). Under the HA+SWE organic amendment, the lowest dry weight of wild weed beets was recorded in the wheat plots (226 g m^−2^), followed by the oat plots (282.45 g m^−2^). Meanwhile, under the HA organic amendment, the lowest dry weight of wild weed beets (280.27 g m^−2^) was recorded in the oat plots (Figure 6).

## 3. Discussion

This study demonstrates that HA and SWE applications can modulate both crop growth performance and weed suppression, eliciting species-specific responses. The HA-SWE treatments consistently produced the largest spike number (Figure 2), greatest kernel weight (Figure 3), grain yield (Figure 4), biomass (Figure 5) and lowest wild beet biomass (Figure 6). These findings align with Nasiroleslami et al. [22], who found that wheat yield was significantly increased following combined HA and SWE application, particularly under increased nitrogen fertiliser at higher rates. They attributed these improvements to physiological and nutritional processes such as the creation of a nutrient sink, new tissue formation, and enhanced photosynthesis [61]. It was also reported that the combination of HA and SWE may improve soil characteristics and promote rhizosphere for nutrient uptake. A similar finding was reported in maize, where organic amendments improved growth and grain yield, potentially through nitrogen use efficiency, micronutrient and macronutrient recovery, phosphorus solubilization and uptake by the plants and enhanced potassium availability for crop plants [62].

Although the study area was irrigated, the weather conditions during the 2021–2022 season likely shaped treatment responses. Extremely low rainfall (2.2 mm) and moderate winter temperatures implied that crop growth depended largely on irrigation. Under these conditions, biostimulants enhancing nutrient uptake and physiological efficiency, particularly HA and HA+SW, would be expected to confer additional benefits, consistent with the increased biomass and yield observed. The variable response to SWE alone may reflect the greater sensitivity of its hormone-mediated effects to environmental fluctuations, even under irrigation.

The increased kernel weight observed in HA-treated barley samples supports the findings of As et al. [60], who reported that growth regulators, such as HA, positively influence source–sink dynamics, particularly during grain filling.

Consistent with our results that the HA organic amendment significantly improved cereal biomass (Figure 6) and grain yield (Figure 5) under semi-arid conditions, a recently published global meta-analysis synthesising 120 field studies and encompassing 479 paired observations [32] revealed that HA increases yield, nitrogen use efficiency and nitrogen uptake in upland cereals and cash crops cultivated in neutral-to-alkaline soils (pH ≥ 6) and regions receiving > 300 mm of annual precipitation remarkably. It further elucidates that HA enhances soil fertility through cation-exchange complexation, stimulation of microbial activity, and formation of organo-mineral associations that improve nitrogen retention and uptake efficiency.

Moreover, the ability of HA to stimulate root growth, improve photosynthetic activity, and accelerate nutrient uptake by modulating phytohormone balance and enzymatic activities [38,39] collectively enhances soil fertility and plant vigour, which likely contributed to the improved cereal performance observed in this study. At the soil surface, HA improves soil structure by improving its cation exchange, water-holding capacities, and reducing the mobility of toxic heavy metals [27,31,42]. These functions are particularly beneficial in the semi-arid conditions of southern Iraq, where soil organic matter content is low and rainfall is restricted. Such mechanisms likely explain the uniform increase in biomass observed in the HA-treated plots. In addition, the stronger response of barley than that of wheat confirms genotype-specific variation in sensitivity to HA [36]. Overall, the uniform response across the cereal crops indicates that these organic amendments can enhance cereal yield.

The HA organic amendment significantly induced weed suppression, as indicated by the reduced biomass of wild weed beets in the barley plots. The influence of HA on crop yield components and wild weed beet biomass can be attributed to its role in improving nutrient uptake and stress resistance by modulating hormonal and redox metabolisms [38,63]. These processes enhanced soil function and barley’s competitive ability, thereby reducing weed growth [61,64].

However, the interaction between crop species and organic amendments indicates that the suppressive effect of the HA organic amendment on wild weed beets varied across cereal species, highlighting crop-specific enhancement of competitive ability. Weed biomass was more greatly reduced in the HA-treated barley plots than in the HA-treated oat and wheat plots (Figure 6), suggesting that oats and wheat require different stimulant treatments to achieve weed suppression levels similar to those observed in barley. The more pronounced effect of HA on the enhanced competitiveness of barley against wild weed beets may be attributed to the rapid early growth and denser canopy of barley, which, together with HA-enhanced vigour, improved its ability to acquire resources and reduce the ecological niche available for wild weed beets [19,20]. This interpretation is further supported by the higher above-ground biomass accumulation observed in barley (Figure 5), although it should be viewed within the limits of the presented data. Further quantification of early growth dynamics and canopy structure is recommended to confirm the underlying mechanism. Similar outcomes were noted by Stybayev et al. [65], who observed that the introduction of cover crops in north-eastern Kazakhstan during spring was an effective way to suppress weeds and enhance forage-crop productivity in the arid steppe soils, highlighting that non-chemical interventions can sustainably improve crop competitiveness by lowering weed pressure while maintaining productivity.

The SWE induced a modest increase in oat spike length and varied effects on spike number in wheat and barley [55,57], consistent with previous findings that SWE may increase kernel weight and crop yield under various nitrogen fertiliser levels [56]. However, inconsistencies in the influence of SWE on crop yield suggest that different cereal species require different optimal doses and application times. Such variation likely reflects the complex bioactive composition (e.g., cytokinins, auxins, betaines, and gibberellin-like compounds), which functions in various ways based on species and developmental stage [39,51].

Recent studies also indicate that the benefits of SWE are amplified when supplemented with nutrient amendments, such as nitrogen [55,56,57]. This is supported by the agronomic practice of synchronising SWE application with crop nutritional status to achieve optimal results.

The combination of HA and SWE reflected the effects of HA alone; however, slight additional improvements in biomass were observed, suggesting potential synergistic interactions. Mechanistically, this can be attributed to the complementary functions of HA (e.g., root and soil enhancement) and SWE (e.g., shoot elongation and reproductive stimulation); hence, a combination of HA and SWE may support both vegetative and reproductive growth [60].

For instance, Al-Saif et al. [59] reported that the foliar application of a combination of HA (2000 mg L^−1^) and SWE (3000 mg L^−1^) on apricot (*Prunus armeniaca* L.) plants grown under arid conditions significantly increased their fruit set, yield, and nutrient content (nitrogen, phosphorus, potassium, calcium, magnesium, iron, zinc, and manganese). Although apricot is a perennial fruit crop, the same mechanism—increasing nutrient uptake and stimulating metabolism—is involved behind the yield-enhancing effects of combined HA and SWE applications on apricots and cereals. Such cross-crop evidence indicates that HA+SWE enhances crop nutrient use efficiency in water- and nutrient-limited environments.

Similarly, Abdel-Sattar et al. [58] observed that combined HA and SWE applications improved the nutrient assimilation, chlorophyll synthesis, carbohydrate accumulation, and fruit yield of mangoes (*Mangifera indica* L.) under arid conditions. Although their [58] work focused on fruit physiology, they confirm that HA and SWE act synergistically to enhance crops’ photosynthetic efficiency and metabolic performance—processes fundamental to spike and grain development in cereals. The parallel mechanisms underlying the enhancement of fruit physiology and improvement of cereal yield substantiate the complementary functions of HA and SWE.

Beyond increasing crop yield, the combination of SWE and HA may reinforce crop vitality via indirect competitive strategies. SWE derived from *Ascophyllum nodosum* enhances leaf area, chlorophyll content, and antioxidant activity of cereals [48,52,54,55,56], resulting in rapid crop establishment and enhanced physiological performance. These traits enhance crop competitiveness by improving resource capture; however, none of the cited studies directly assessed the effects of HA and SWE on weed suppression, rhizosphere microbial community composition, or allelopathic interactions between crops and weeds.

Evidence for weed suppression via combined HA and SWE applications in cereals remains limited. Nevertheless, HA+SWE organic amendment has been shown to enhance foliar nutrient content, chlorophyll concentration, and yield in fruit crops [58,59], supporting the possibility of enhancing crop vigour and competitiveness under semi-arid conditions.

As the field experiment was conducted under the semi-arid conditions of southern Iraq, characterised by low precipitation and limited soil organic matter, the results of this study suggest biostimulants as sustainable substitutes for chemical herbicides and fertilisers. Meanwhile, despite being targeted, SWE’s dose- and time-manipulative response underscores the imperative of site-specific calibration of dose, time and crop selection. Such biostimulant application procedures fit within integrated weed management [15,66], enabling stable cereal yield in semi-arid environments.

The interpretability of the present findings is framed by the experimental context. Since the trial was conducted at a single semi-arid site and at fixed HA and SWE rates, the observed responses reflect performance under a single set of climatic and edaphic conditions rather than the full range of environments whereby cereals are cultivated. Similarly, wheat, barley, and oats responded to a single dominant weed competitor, indicating that crop weed dynamics may differ in alternative community compositions. These constraints do not undermine the trends identified here but clarify that the mechanisms proposed, particularly the differential enhancement of crop vigour and weed suppression, should be considered under contrasting environmental conditions and management intensities to assess their stability.

## 4. Materials and Methods

### 4.1. Site Description

A field experiment was conducted during the 2021–2022 winter season at the Forest Nursery Farm in Amarah City, southeast Iraq (31.88898 °N latitude and 47.08025 °E longitude). The study site was 12 m above the mean sea level. Prior to planting, composite soil samples were collected from multiple locations across the experimental field to analyse key soil physical and chemical properties using the standard procedures outlined by Black [67] and Page et al. [68].

**Figure 7:**
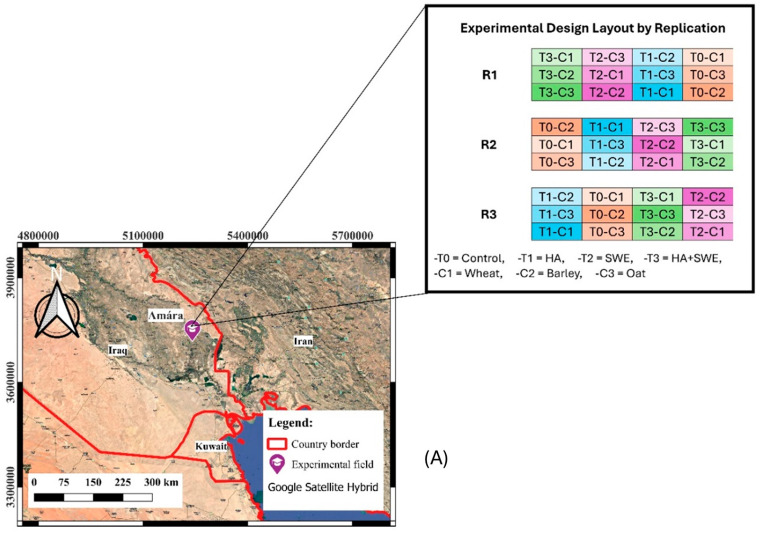

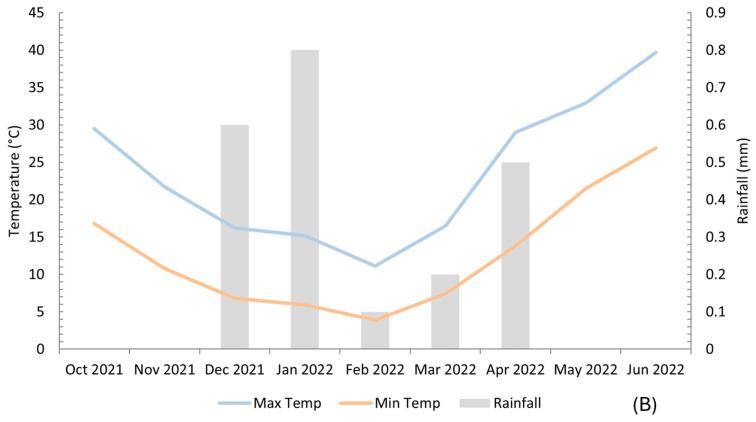
Overview of the experimental field and data collection procedure. (**A**) Geographical map of the study area and field layout showing different combinations of treatments and cereal crop species. To represent the same treatment, blocks R1, R2 and R3 are of the same colour. (**B**) Monthly maximum and minimum air temperatures and rainfall at the experimental site in Amarah, Iraq, during the 2021–2022. Temperature values represent monthly means, while rainfall values represent total monthly precipitation.

Amarah City has a hot desert climate, characterised by extremely hot, dry summers and cool, wet winters. The temperature ranges between −1 °C and 26 °C from December to February and 33–40 °C from March to May, reflecting its characteristic dryness and significant thermal fluctuation throughout the year. The climatic characteristics of the study site are presented in Figure 7. The mean temperature during the growing season was approximately 18.1 °C, and the total rainfall was approximately 2.2 mm. Figure 7B shows the monthly average temperature and precipitation during the study period. The average temperature decreased from October to February, then increased from March to June. In contrast, the average precipitation exhibited a fluctuating pattern: it steeply increased from October to January and from March to April, then drastically declined from January to February and from April to June [69,70].

Based on texture, the soil in the experimental field was classified as silty clay loam. Soil pH was 7.82, determined according to the method described by Page (1982) [68], organic matter content was low (0.98%) and nitrogen, phosphorus and potassium concentrations were 28.50, 16.25 and 21.00 g·kg^−1^, respectively, expressed on dry soil basis (Table 1). Other soil physical and chemical properties are listed in Table 1.

### 4.2. Experimental Design

The experiment was conducted during the 2021–2022 winter season by applying a randomised completely blocked design (RCBD) using a split-plot arrangement with three replicates. A summary of the crop characteristics, plot layout, and experimental design is provided in Table 2. Each block was divided into four whole plots; then, each whole plot was further subdivided into three subplots. Each subplot had an area of 4 m^2^, consisting of eight crop lines with 25 cm row spacing. The whole plots were randomly assigned to four experimental setups corresponding to biostimulant (organic amendment) application: control (no organic amendment), HA (5 mL·L^−1^), SWE (Ascophyllum nodosum, 3 mL·L^−1^) and a combination of HA and SWE (HA+SWE). Meanwhile, three cereal crops that are adaptable to the semi-arid environments in southern Iraq, a Spanish wheat variety (Triticum aestivum cv. Ibaa 99), barley (Hordeum vulgare cv. Bohoth 244) and an oat variety that has adapted to the study site (Avena sativa cv. Shifa) were randomly planted across the subplots on 10 December 2021 (Figure 7A).

The plants emerged at 10–14 weeks after sowing and were irrigated and fertilised regularly during the growing season. The organic amendments were applied approximately 60 days after sowing (i.e., upon reaching the tillering stage). HA and SWE were applied in the early morning under cool conditions (approximately 15–18 °C) and minimal wind, ensuring proper spray deposition, reduced evaporation, and uniform absorption. Standard agronomic practices, including tillage, irrigation, fertilisation, and pest control, were applied uniformly across all plots to ensure consistent growth conditions (Table 2). The most prevalent weed species in the field was wild weed beets. Other weed species emerged sporadically and were cleared by hand during the initial crop cultivation to ensure that wild weed beets served as the primary competitor of the cereal crops.

### 4.3. Measurements

The spike length, spike number, kernel weight, grain yield and crop biomass of the cereal crops as well as wild beet biomass were recorded at the harvest stage (about 150 days after sowing). The sample size was 0.0625 m^2^ per subplot (Table 2). The spike number and wild beet biomass were calculated and converted to number and gram per square meter, respectively, while grain yield and total biomass were converted to tons per hectare (t·ha^−1^). The samples were dried at 75 °C for 72 h and then reweighed to determine the dry biomass to the nearest milligram [71].

### 4.4. Statistical Analysis

The collected data were statistically analysed using R i386 software v.3.5.3 [72]. Two-way ANOVA was 566 performed to examine the effects of crop species (barley, oats and wheat) and soil amendment treatments (control, HA, SWE and HA+SWE) on aboveground crop biomass. The significant differences among soil amendment treatments were separated at the 0.95% confidence interval [72,73]. A basic linear model was performed with the two-way ANOVA to examine the effects of crop species (barley, oats and wheat) and soil organic amendment treatments (control, HA, SWE and HA+SWE) on aboveground crop biomass. The source of variances and statistical values of all parameters are listed in Table 3.

## 5. Conclusions

In this study, the synergistic effects of HA and SWE, which are known biostimulants, on cereal crop competitiveness with wild beets were determined. HA and HA+SWE consistently improved crop biomass and yield components, whereas the effects of SWE alone varied and appeared species dependent. The interaction between cereal crop species and biostimulant treatments highlights the need for crop-specific weed management strategies. These findings provide a solid foundation for recommending tailored amendment regimes to enhance crop competitiveness, suppress weeds and improve the yield and quality of cereal cropping systems. Nonetheless, multi-site and multi-season experiments should be conducted in the future to evaluate the consistency of HA and SWE effects under diverse conditions. Field experiments involving a wider range of doses, varying application schedules, and combinations with different fertilisers will enable management optimisation. Future research combining physiological and molecular studies is required to identify the underlying mechanisms underlying the responses of the crop species included in this study. As our findings revealed, wheat, barley, and oats did not respond equally to HA and SWE organic amendments; thus, it can be postulated that the differences are attributable to variation in hormonal regulation, nutrient transport, or the expression of stress-related genes. The study of these molecular pathways will aid in understanding the mechanisms whereby biostimulants such as HA and SWE regulate crop performance and weed suppression.

## Figures and Tables

**Table 1:** Significance levels from analysis of variance for crop parameters and wild beet biomass.

Parameters	Factors		
	Crops	Treatments	Crops × Treatments
Spike length	***	NS	*
Spike number	NS	**	*
Kernel weight	***	**	*
Crop grain yield	***	**	NS
Crop biomass	**	*	NS
Wild beet biomass	*	**	**

* Significant at *p* ≤ 0.05; ** Significant at *p* ≤ 0.01 and *p* < 0.01; *** Significant at *p* < 0.001; (NS) non-significant.

**Table 2:** Summary of continuous and categorical variables used in the field experiment.

Category	Variable (Unit)	Value or R
Environmental	Location (°N, °E)	31.88898 °N, 47.08025 °E
	Elevation (m.a.s.l.)	12
	Soil texture	Silty Clay Loam
	pH (1:1H_2_O)	7.82
	Organic matter (%)	0.98
	Nitrogen, N (g·kg^−1^ dry soil)	28.50
	Phosphorus, P (mg·kg^−1^ dry soil)	16.25
	Potassium, K (mg·kg^−1^ dry soil)	21.00
	Calcium, Ca^2+^ (mmol·L^−1^)	1.78
	Magnesium, Mg^2+^ (mmol·L^−1^)	0.24
	Sodium, Na^+^ (mmol·L^−1^)	8.95
	Potassium, K^+^ (mmol·L^−1^)	0.17
	Sulphate, SO_4_^2−^ (mmol·L^−1^)	2.50
	Bicarbonate, HCO_3_^−^ (mmol·L^−1^)	0.80
	Chloride, Cl^−^ (mmol·L^−1^)	8.62
	Monthly maximum temperature (°C)	11.3–38.7 (October–June)
	Monthly precipitation (mm)	0.0–0.8 (October–June)
	Mean seasonal temperature (°C)	18.1
	Total seasonal rainfall (mm)	2.2
Crop and experimental design	Experimental design	Split-plot design integrated within a randomised completely blocked design
	Replications (count)	3
	Crop species	Wheat, Barley, Oat
	Seeding rate (kg·ha^−1^)	140
	Inter-row spacing (cm)	25
	Plot size (m^2^)	0.0625
	Weed sampling date	17 April 2022
	Harvest date	23 April 2022
Management	Tillage method	Mold-board plough
	Irrigation	As needed
Treatments and inputs	Treatments	Control, HA, SWE, HA+SWE
	Application method	Manual backpack sprayer
	Application date	7 February 2022
	HA composition	80% Humic + Fulvic Acid, 8.5% K_2_O (soluble)
	SWE composition	20% *Ascophyllum nodosum*, 4% alginic acid, 5% mannitol, 1.25% K_2_O
	Combined HA+SWE	HA-SWE of HA and SWE at respective rates

**Table 3:** Two-way ANOVA results for the effects of crop species, organic amendments, and their interaction on cereal growth parameters. SOV, DF, SS, MS, F-values, and *p*-values are shown.

Parameter	SOV	DF	Sum of Sq.	Mean Sq.	F Value	*p* Value
Spike Length	Replicates	2	3.7	1.9	1.782	0.1967
	Crop Spp.	2	1292.3	646.1	615.703	<2 × 10^−16^
	Organic Amendments	3	1.2	0.4	0.393	0.7592
	Interaction	6	17.4	2.9	2.758	0.0443
	Residuals	18	18.9	1.0		
Spike Number	Replicates	2	109,278	54,639	2.879	0.0822
	Crop Spp.	2	32,363	16,182	0.853	0.4428
	Organic Amendments	3	303,043	101,014	5.323	0.0084
	Interaction	6	388,756	64,793	3.414	0.0199
	Residuals	18	341,567	18,976		
Kernel Weight	Replicates	2	22.1	11.0	2.229	0.1365
	Crop Spp.	2	1862.3	931.2	187.984	8.68 × 10^−13^
	Organic Amendments	3	90.5	30.2	6.087	0.0048
	Interaction	6	79.4	13.2	2.670	0.0495
	Residuals	18	89.2	5.0		
Grain Yield	Replicates	2	191,854	95,927	5.892	0.0129
	Crop Spp.	2	803,426	401,713	24.672	1.81 × 10^−5^
	Organic Amendments	3	336,220	112,073	6.883	0.0039
	Interaction	6	55,728	9288	0.570	0.7478
	Residuals	18	244,228	16,282		
Crop Biomass	Replicates	2	293,753	14,687	0.655	0.5322
	Crop Spp.	2	4,496,075	2,248,037	10.021	0.0013
	Organic Amendments	3	3,070,403	1,023,468	4.562	0.0161
	Interaction	6	3,195,962	532,660	2.375	0.0751
	Residuals	18	3,813,509	224,324		
Wild Beet Biomass	Replicates	2	11,059	5530	0.835	0.4498
	Crop Spp.	2	72,850	36,425	5.503	0.0137
	Organic Amendments	3	111,783	37,261	5.630	0.0067
	Interaction	6	206,156	34,359	5.191	0.0029
	Residuals	18	119,139	6619		

## Data Availability

The data presented in this study are available on request from the corresponding author. The data are not publicly available due to privacy or ethical restrictions.

## Abbreviations

The following abbreviations are used in this manuscript:

HA	Humic acid
SWE	Seaweed extract
(HA+SWE)	mixture of HA and SWE
ANOVA	Analysis of variance
